# Evaluating the efficacy of computer games-based learning intervention in enhancing English speaking proficiency

**DOI:** 10.1016/j.heliyon.2024.e36440

**Published:** 2024-08-16

**Authors:** Omar Al-Jamili, Musharraf Aziz, Fathey Mohammed, Abdullah Almogahed, Abdulwadood Alawadhi

**Affiliations:** a Department of Computers and Information Technology, Faculty of Engineering and Computing, University of Science and Technology, Yemen; b Department of Applied Linguistics, Faculty of Administrative and Human Sciences, University of Science and Technology, Yemen; c Department of Information Technology, Faculty of Engineering and Information Technology, Taiz University, Taiz, Yemen; d Department of Software Engineering, Faculty of Engineering and Information Technology, Taiz University, Taiz, Yemen

**Keywords:** Action research, Computer game-based learning, English speaking proficiency, English conversation practice game, Gamification in education

## Abstract

The use of learning games in education, particularly for second language (L2) acquisition, has gained significant traction recently, establishing game-based learning as a notable academic discipline. This study examines how computer game-based learning influences ESL undergraduates’ speaking abilities, comparing traditional teaching methods with game-based teaching techniques. The study employed action research utilizing a control-experimental groups technique with a sample of 60 learners. Data were collected through observation sessions, interviews, as well as pre-tests and post-tests on English speaking skills. Upon comparing the scores of the control and experimental groups, the experimental group showed greater improvement in speaking skills. This study provides significant insights into the area of game-based learning using computers, particularly among international students in ESL contexts.

## 1 Introduction

Game-based foreign/second language learning has captured the attention of 21st-century education stakeholders due to the pressing demand to produce proficient critical thinkers and language users. This contemporary methodology has been widely embraced, especially in developed educational sectors, and is now recognized as a distinct field of study and research. Game-based learning is defined as a joyful cognitive activity governed by academic guidelines aimed at effectively achieving a significant number of educational outcomes [1]. The technique can broadly be divided into computerized and non-computerized forms involving learning games.

Some researchers [2,3] argue that integrating learning content with digital games can be an efficient way to engage students in targeted and enjoyable learning. It provides learners with opportunities to acquire academic skills and desirable behaviors in a friendly manner. Moreover, it captures learners’ attention and motivates them towards acquiring desirable skills. In this way, learners are cognitively highly engaged with the learning content, a goal sometimes not achievable with traditional ‘dry and drab’ learning methods. They are less popular, as compared to synergetic game-based learning, because of their conventional focus on teacher-oriented lecture-based techniques that are usually limited to textbooks, inclining towards didactic approaches [4].

In recent years, game-based learning has gained the status of an innovative instructional strategy in academia. It is also used to enhance higher-level learning across various disciplines, such as mathematics, language, business, health, computing, nutrition, crisis management, and tourism [5,6]. Currently, it serves as a solid theoretical foundation for instilling effective learning with practical outcomes. Similarly, the field of L2 learning is considered one of the significant beneficiaries of game-based learning theory [7].

English is recognized as a global language as it bridges native and non-native speakers, as well as speakers of other languages from different parts of the world. Several authors [8,9] noted that English is the most widely used and standard medium of communication among people from diverse cultural, ethnic, and social backgrounds. Furthermore, a vast amount of knowledge is globally accessible in the English language, necessitating proficiency at all levels, particularly at the university level. However, in many ESL, international students struggle to meet the required standards of English to continue their academic pursuits and cultural integration. Although all four English skills are essential, speaking skills are considered crucial for international students since they are not only a means to access knowledge but also to communicate successfully with other learners from diverse backgrounds.

University Utara Malaysia (UUM) in Sintok, Malaysia, is an accredited international university with a considerable number of international students. At UUM, learners whose first language is not English are required to pass either the TOEFL (with 500 points) or the IELTS (with Band 6.5) for admission eligibility. If candidates do not meet these test scores, they must enroll in a compulsory Intensive English Course (IEC) to gain admission to their desired school. However, the UUM Language Centre reported that 60 % of international students required to take the Intensive English Course failed in Semester A222 in 2022. Additionally, the weakest English language skill among these learners was speaking. In a university where students come from diverse backgrounds, English speaking skills are the most significant tool for communication, acculturation, and learning [10]; therefore, this result raised severe concerns among both provider and consumer stakeholders at UUM.

In response to the above concern, the researchers aimed to investigate whether an intervention based on computer games could improve the current learning performance of these students. The study hypothesized that one of the significant shortcomings in the language learning of these students is the conventional and unengaging methods of language teaching. Moreover, these methods can sometimes be more demanding than simple immersion and motivation techniques such as suggestopedia and the direct method. Generally, in developing educational sectors, teacher-centered and rote learning approaches hinder learners' satisfactory performance. Thus, the current study aimed to determine the effect of computer game-based learning on the English-speaking skills of these learners at UUM. Based on this purpose, the following hypothesis was formulated:

H1 *Computer game-based intervention in ESL speaking skills increases the learner's performance.*

## 2 Review of related literature

### 2.1 Challenges in speaking skill acquisition

It has been noted that non-native English learners frequently face significant challenges in the target language (TL), particularly in the four primary skills: listening, speaking, reading, and writing. Speaking skill difficulties are often due to a lack of appropriate participation, stemming from factors such as inhibition, L1 interference, repression, demotivation, and disinterest [11].

#### 2.1.1 L1 interference

In many academic contexts, learners share a common L1, which they prefer for several reasons. Firstly, it is less demanding. Secondly, speaking in a foreign language feels unnatural without sufficient exposure and practice, leading to a reliance on the mother tongue. Thirdly, the absence of opportunities to use the TL, inadequate practice, and a lack of feedback result in L1 interference when attempting to speak in the TL. Additionally, the prevalence of L1 in social situations makes it challenging for learners to utilize the TL, particularly for those with minimal exposure, resulting in greater L1 interference [12]. Lastly, these linguistic and learning challenges are compounded by a lack of engaging teaching methodologies and content.

#### 2.1.2 Lack of participation

Turn-taking is an essential aspect of speaking. Large class sizes, especially in the context of developing TL teaching, limit individual speaking opportunities. When teachers attempt to provide these opportunities, constraints such as limited speaking time and class duration act as barriers. This issue is further complicated by the tendency of some learners to dominate while others remain silent. The majority of L2 learners cannot overcome this inhibition without external motivation, stimulated interest, and practice. Game-based techniques and digital engagement can address these challenges in situations where learners struggle to achieve satisfactory L2 speaking skills [13].

#### 2.1.3 Repression

Speaking skills require real-time engagement in communicative situations, unlike reading, writing, and listening. Learners often hesitate to speak in the TL due to nervousness, fear of negative evaluation, limited vocabulary, and a lack of sentence construction strategies. The absence of engaging practice methods exacerbates these barriers [14].

#### 2.1.4 Lack of motivation

Uninspiring teaching methods can lead to learner disengagement, content overload, and boredom. Learners who struggle may feel less motivated to speak in the TL, especially when the content and context are uninteresting. Without timely intervention, this demotivation can grow, negatively impacting performance in speaking skills, which requires immediate reflection. Games can be particularly effective due to their entertaining nature and ability to engage L2 learners for extended periods without boredom or frustration [15].

### 2.2 Games in the classroom

A game is defined as a challenge involving skill, effort, and luck intended for entertainment [16]. It can also be seen as engaging play within a competitive environment, invoking cognitive engagement and stimulation. Researchers [17] identify critical components of using games for learning, including enjoyment, structured procedures, rules, and rewards. Games offer a welcome break from traditional classroom routines. Researchers [18] suggest that classroom diversions are more than mere entertainment; they are cherished by both learners and instructors for transforming learning into an enjoyable experience, thus enhancing motivation and skill acquisition. Additionally, game-based learning promotes socialization, teamwork, collaboration, and appreciation among learners, positively influencing performance and classroom atmosphere.

### 2.3 Advantages of games in second/foreign language classrooms

The significant benefits of incorporating games in the classroom, such as breaking the monotony, stimulating motivation, fostering collaborative skills, providing exposure to communicative scenarios, and enhancing critical thinking, are recognized [19]. Game-based activities have been shown to have a remarkably positive impact on learning. For example, two researchers [20,20] developed a digital game to improve English learning in request-making skills, finding that it positively influenced learners' attempts and engagement. Similarly, it has been observed that computer game-based learning aids in vocabulary acquisition and skill transfer among foreign language learners [21].

Games not only engage learners but also enable teachers to transform the classroom into a dynamic and enjoyable learning environment. This sense of achievement can inspire teachers to explore innovative methodologies. Games can also serve as assessment tools, providing a more accurate reflection of language proficiency than traditional tests. Moreover, games are versatile and can be used to enhance all language skills, including listening, speaking, reading, and writing.

## 3 Methodology

The research adopted an action research method with mixed-method data collection approaches. The quantitative aspect involved pre and post-tests to measure the improvement in English speaking skills among participants using a Computer Games-Based Learning (CGBL) approach. The qualitative aspect included interviews to gather participants' feedback on their learning experience, motivation levels, and perceived challenges. The sample consisted of non-native English speakers from diverse backgrounds enrolled in an English language learning program.

The present study utilized action research to leverage game-based learning theory. Action research is defined as a systematic process of improvement whose primary outputs are innovative teaching and evaluation materials, methodologies, and pedagogical standards [22]. This study posited that using computer games to teach ESL speaking skills would enhance learner performance and yield higher-quality learning compared to traditional, non-gaming environments.

### 3.1 Research design

The study employed a sequential research design with two cycles, aiming to compare the outcomes of two distinct classroom groups labeled as control and experimental groups. The target population was international students at the University of Utara Malaysia who were enrolled in an intensive English course. It is a compulsory course for every international student who needs to start postgraduate courses, as English is the sole medium of instruction in the university except for the specialized courses in the Malaysian language. For research purposes, the authors were allowed to access one class (*n* = 30), of which an experimental group (*n* = 15) and a control group (*n* = 15) were formed. Participants were randomly selected from an Intensive English Language course at UUM to minimize selection bias. They were deemed homogeneous, having been thoroughly vetted for the course by the university. For random sampling, the digital list of students’ matric numbers, provided by the class instructor, was used. In one round, the computer randomly selected 15 participants for experimentation. Of the remaining numbers, a control group was formed. Digital sorting was applied to sustain the probability of selection and reduce bias. Table 1 presents the demographic information of the participants:

**Table 1:** Demographic information of the samples.

	Age Range	Proficiency Level	Gender	No. of Samples
Experimental Group	18–20 years	High School English	Female	8
	18–20 years	High School English	Male	7
Control Group	18–20 years	High School English	Female	9
	18–20 years	High School English	Male	6

Table 1 depicts that the learners were a homogenous group because of the careful selection by the university. They were given admission to the undergraduate level education therefore, the ages ranged between 18 and 20 years. They had completed their high school education, and the related requirement of English was satisfied. Further, both female and male learners were enrolled in Intensive English Course classes. Table 1 shows that 17 females and 13 male learners were randomly selected using digital lists on computer.

**Fig. 1:**
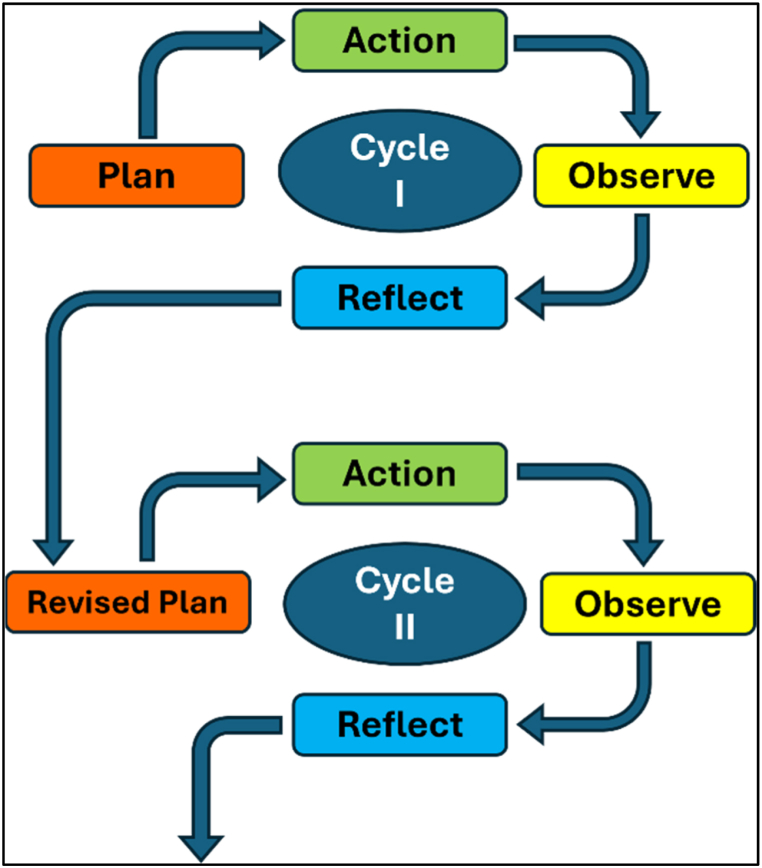
Action research model [22].

The experimental group used a computer gaming application for English-speaking practice, while the control group received conventional ESL instruction. As a classroom action research (CAR) project, the goal was to enhance English speaking skills by involving participants in a learning environment where they could plan, implement, evaluate, and reflect on their language learning through the use of a computer game application. In CAR, the dynamics of discussion and collaboration among participants are crucial to the research's success and quality [23,23]. Therefore, participants were encouraged to engage in dialogue and collaboration during the learning process. Fig. 1 outlines the research procedures.

Fig. 1 illustrates the operational dynamics of the CAR model by solving problems and improving practices through continuous feedback loops. It consists of two cycles, each with four phases: Plan, Action, Observe, and Reflect. Initially, a plan is formulated to address an identified problem, which is then implemented (Action). The outcomes are monitored (Observe), and the results are analyzed (Reflect) to assess effectiveness. This model is considered highly operable because of its double-check nature and variety of measures in the form of critically reflecting on the results of the first plan or cycle and then moving ahead based on observations and practical insights.

### 3.2 Research process

**Fig. 2:**
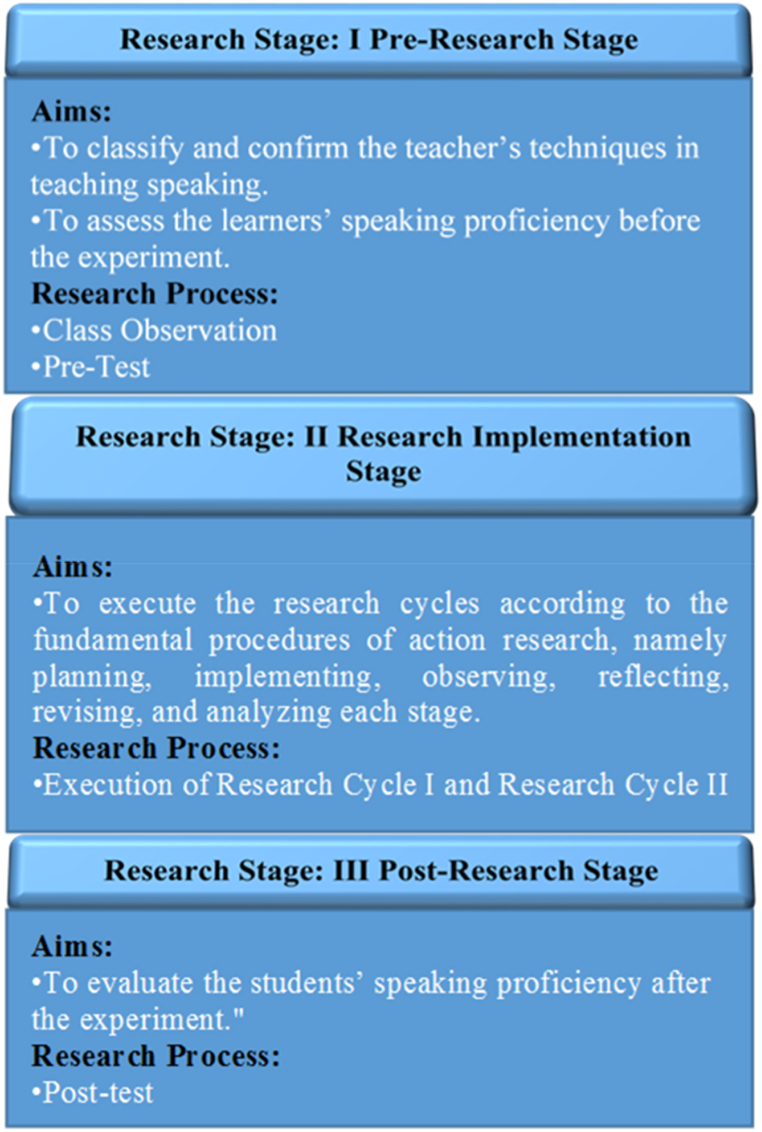
Research process.

The research process of this study was comprised of three stages commonly associated with action research: the pre-research stage, the research implementation stage, and the post-research stage. Fig. 2 outlines the objectives of each stage as they pertain to the current study.

### 3.3 Data collection methods

The data for the current study were collected through class observation, interviews, a pretest, and a post-test of English-speaking skills. These methods are elaborated on below:

#### 3.3.1 Semi-structured observation form

To facilitate the research, an observation form was developed, drawing from various data sources. This form was crafted through a series of steps. Initially, seven questions aligned with the research objectives were devised. These questions underwent a review for content and construct validity by experts in the relevant field. Based on their feedback, modifications were made to the wording of the questions, leading to a refinement in their number. Ultimately, the form consisted of four well-defined questions, which were again reviewed and approved by the domain experts.

A pilot study was then conducted to assess the clarity and comprehensibility of the observation form. This preliminary study involved six students (three female and three male) from the same participant pool as the main study. The feedback from this pilot study was instrumental in identifying and rectifying any ambiguities in the form.

The finalized observation form focused on three key areas: the execution of the teaching process, the student's emotional responses to the course, and the feasibility of the action plan. The researcher played a pivotal role in developing this data collection tool. Throughout the research, a total of eight observation forms were gathered, providing valuable insights for the study.

In this regard, field notes are a significant instrument since they permit an in-depth comprehension of what happens in the selected environment. For pertinent observation, factors such as collaboration between the researchers and respondents, the exercises created in the classroom, the verbal instructions, the nonverbal cues, and cooperation are significant.

The field notes in the current study were adopted from related studies [24,25] and as an apparatus to record what was observed in every session in terms of the physical environment, listening materials, listening media, students' behavior, and teaching methods.

#### 3.3.2 Semi-structured interview form

This data collection instrument was designed to capture participants' expectations and experiences related to using digital games in the classroom. The development of this tool involved a structured process. Initially, five questions were crafted, aligning with the research objectives. These questions were then reviewed by three domain experts for content and construct validity. Based on their input, an additional question was added, bringing the total to six.

To verify the suitability of the questions for the target audience, a preliminary study was carried out involving six students (comprising an equal number of three females and three males), who mirrored the demographic of the primary study's participants. This preliminary study helped refine the questions, making them clearer and more targeted.

The final version of the semi-structured interview form consisted of six questions. These questions delved into various aspects, including the learning topics within the research, participants' views on the research process, their opinions about the control class, and their thoughts on digital gamification. To gain comprehensive insights, 12 interviews were conducted, both before and after the digital game was used in the classroom.

#### 3.3.3 Testing the game

A test can be characterized as a method for measuring a learner's performance, knowledge, or skills in a given learning dimension [26]. In the current study, the respondents were administered a pre-test and post-test with the objective of determining the level of their understanding of the lessons given to them using a computer-based speaking skill game referred to as English Conversation Practice (ECP), which is also available as a commercially available free digital app. The rubric used for comparing the level of improvement in the learners was developed by previous researchers [27]. The ECP game was selected to be used as the intervention measure. This game was selected for three reasons: (a) The genre of the game was functional and inclusive in terms of English speaking; (b) It offered a variety of contents and communicative situations that could be used in combination with English-speaking lessons. It is because both the ECP game and English speaking skill lessons were based on those language functions that are generally required to be learned in ESL speaking classes at the undergraduate level, and (c) The interface of the game was user-friendly and the learner could use this game as a tool for self-regulated learning even after the research was completed. The ECP game, in this study, was used at the intervention stage. It is an interactive game that was administered face-to-face during the intervention classes. This game has several speaking lessons that are based on frequently used language functions such as socializing, greeting, misunderstanding, congratulating, and calling, etc., presented with a speaking interface. Adult learners can also use it as a self-learning tool once they are well-introduced to it in the classroom.

The process of research was conducted in two cycles, each of which was one week. In each week, the researchers, for the research purpose, were allocated five classes by the university. Hence, four classes in each cycle were for observation and intervention whereas the last class was for the tests.

### 3.4 Data analysis

It is asserted that the collected data should be appropriately analyzed to arrive at pertinent conclusions of the research [28]. Data analysis refers to synthesizing, sifting, organizing, and summarizing the data using relevant techniques. Thus, in the current study, the data collected by the pre-test and post-test were analyzed by conducting frequency statistics using SPSS v. 26.0. The data from the semi-structured observations underwent descriptive analysis, a method chosen to methodically detail the implementation process. For the semi-structured interview form, content analysis was employed to provide an in-depth presentation of the data.

The first phase involved a macroanalysis of all data collected during the implementation. The findings from this macroanalysis were regularly reviewed by a validity committee comprising three domain experts on a weekly basis. This committee played a crucial role in both completing the action research cycle and refining the analysis process.

In the second phase, after the application period concluded, a microanalysis was conducted on all the data. This involved transcribing the data, a task facilitated by the use of qualitative data analysis software NVivo 1.0. To ensure accuracy in transcription, the results were reviewed by a domain expert, who confirmed the consistency between the original data and the transcribed version.

Following this, detailed analyses were performed. The outcomes of these analyses were then cross-referenced with the findings from both the initial macroanalysis and the subsequent microanalysis. Additionally, another domain expert independently coded the same data. The coding consistency between the two experts was found to be 90 %, a rate deemed satisfactory based on the standard indicated by previous researchers [29]. This high level of coding reliability underscored the robustness of the analysis.

Regarding the study's validity and reliability, all data gathered during the research were analyzed in their original form without alterations. The study's validity and reliability were consistently verified at each stage by a validity committee from the start of the research process. To further enhance the study's validity, we adopted specific headings based on a previously validated framework [30,30].

### 3.5 Research implementation and findings

Based on the methodology elaborated in the action research model developed by O'Brien [22], two research cycles were conducted in this study:

#### 3.5.1 Planning of Cycle 1

The English teacher, being one of the authors, was involved in the research planning. This assisted in learning about the students' interests and selecting the appropriate lesson for them, keeping in view the selected game. Further, the students were not guest-conscious because of the presence of their teacher in the classroom. It aided the research process and reduction of anxiety among the students. The researcher teamed up with the teacher to examine the generic type of the lesson and materials to evaluate if they suited the syllabus. The selected lesson plan was about socialization in which speaking at both family and friends' meetings were included. To evaluate students’ current speaking skill level, a pre-test was designed in the form of a dialogue.

#### 3.5.2 Action and observation of Cycle 1

**Fig. 3:**
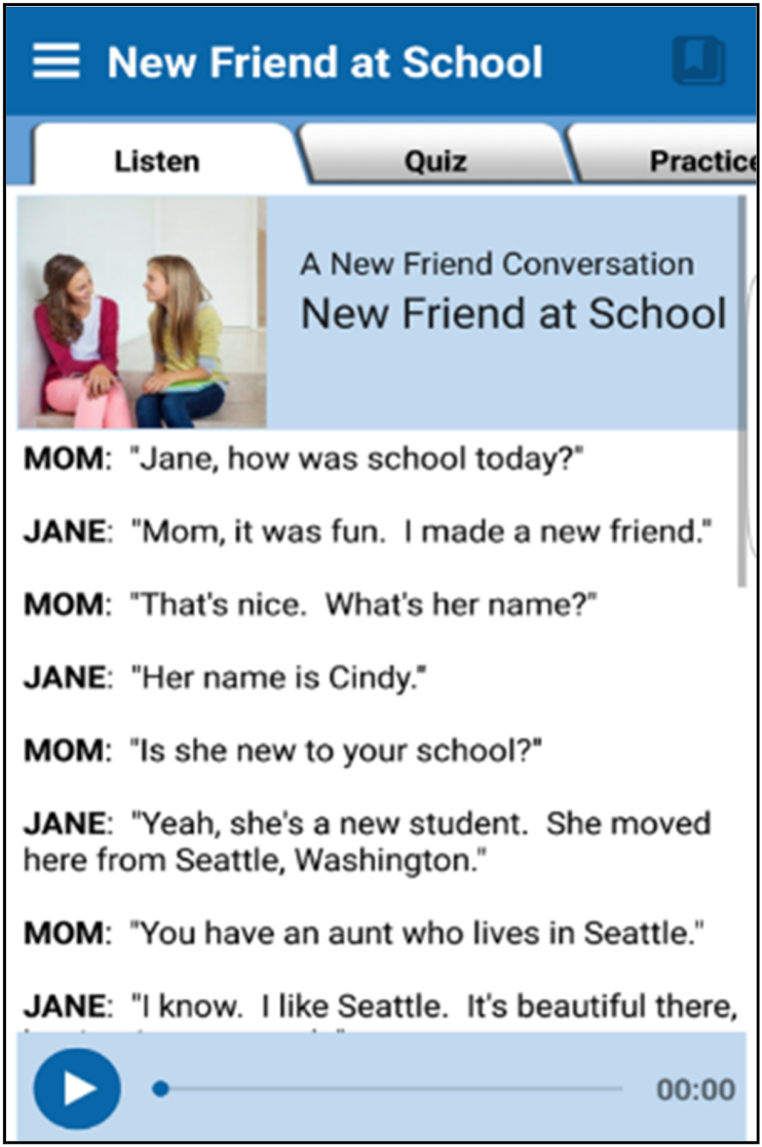
Lesson 1, ‘new friend at school’.

The pre-test was administered to both experimental and control groups, before applying the ECP game intervention. This step was taken at the pre-research stage to collect data on the respondents' proficiency before applying the game-based learning strategy. The skills that were measured in the pre-test were fluency, accuracy, clarity, pronunciation, and content, as recommended by Purnawam et al. [27]. Subsequently, the ECP game was administered to the respondents in the experimental group (*n* = *15), While the control group (n* = *15) was taught through* regular university exercises. In the ECP intervention, the target skills and the expected learning outcomes consisted of the participants’ ability to talk about their family and friends comprehensively. The application stage involving the game had two steps:

• Applying a listening activity as an input action before the respondents were required to speak.
• Using ECP to assist the respondents in speaking by engaging them. Fig. 3 illustrates the lesson that was executed in this cycle.

#### 3.5.3 Reflection on Cycle 1

The researcher's meeting with the students in Cycle 1 took place in the presence of the teacher. The collaboration with the teacher was arranged to reduce any anxiety the learners might feel due to the presence of a guest. Consequently, they felt supported and less inhibited by the presence of a new individual in the classroom. The environment remained familiar to them because of their teacher's involvement. The respondents did not express any displeasure or discomfort with the presence of the researchers.

During the administration of the lesson, some respondents were motivated to perform, while others seemed unable to appreciate this session. However, the majority were willing to speak, though they did not appear confident in their English-speaking abilities.

Based on the findings of the pre-test, the researchers found that seven respondents had a poor level of speaking skills. This outcome urged the researchers to assist them in improving their performance. Additionally, the researcher identified three prominent speaking problems:

• Almost all learners were hesitant to initiate conversation.
• Some learners tended to speak in a low tone, probably due to inhibition and fear of being ‘incorrect’ while attracting attention.
• Most learners had weak English pronunciation, which was a barrier to comprehension.

To enhance performance in these aspects, the teacher randomly asked the learners to perform the conversation. This approach motivated other learners to participate and perform. The researcher observed that the respondents' behavior towards participation somewhat improved, due to practice and motivation through the game. Thus, in the second session, the students seemed more dynamic and willing to participate than before, because of the anticipation of amusement and game assistance. They were ready to learn through the game and willing to form groups and practice. The majority of the students attempted to communicate in English when asked before and during the execution of the ECP.

#### 3.5.4 Findings of Cycle 1

• The learners were initially hesitant to communicate; however, the briefing about using the game for learning speaking skills seemed to engage them with the expectation of upcoming relief and enjoyment.
• The prospect of a diversion from the ordinary speaking class motivated the learners to learn.
• The ECP game was effective in empowering the learners and appeared to increase their confidence and willingness to speak.
• The researcher found that the learners required more time to understand the rules of the game than was allotted. They needed more practice.
• The learners' conversations on the given topic showed signs of improvement in terms of vocabulary, accent, and sentence construction.

#### 3.5.5 Planning of Cycle 2

In light of the findings from Cycle 1, the researcher selected an interesting conversation in the ECP game as the next speaking lesson. To address some of the difficulties observed during Cycle 1, the researcher attempted to overcome them by:

• Choosing a lesson that addressed the learners' current needs in their university life.
• Stratifying the input listening activity into groups and pairs before asking the learners to speak.
• Repeating the pronunciation of difficult words several times without highlighting the learners' mistakes.

#### 3.5.6 Action and observation of Cycle 2

**Fig. 4:**
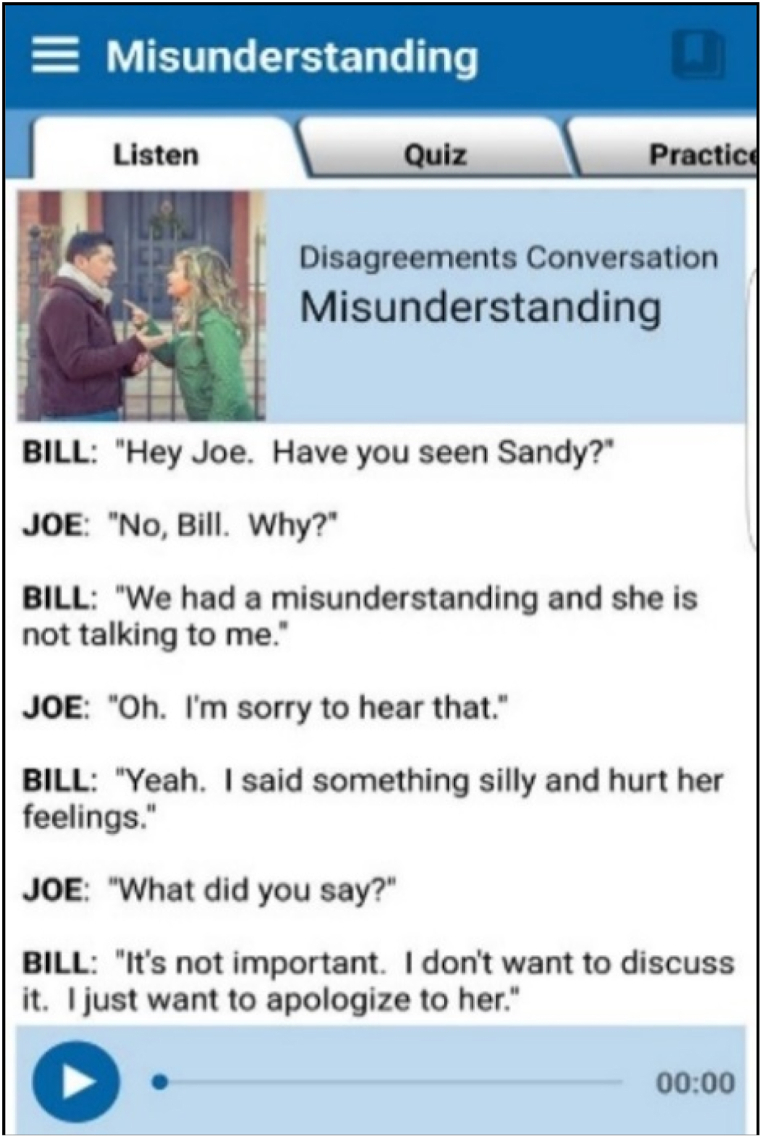
Lesson 2: Misunderstanding.

The participants practiced a topic that involved a misunderstanding between two good friends, both adults, who had some disagreement. The topic, as applied in the ECP, is shown in Fig. 4.

#### 3.5.7 Reflection on Cycle 2

The researcher developed Cycle 2 based on the reflections from Cycle 1. It was observed that the interesting topic and practice on pronunciation engaged the learners more than before. They overcame most of the speaking problems related to vocabulary and pronunciation that were observed in Cycle 1. Moreover, sufficient time was allocated for practice in this cycle, which had a positive effect. It was also observed that practice helped the learners understand the rules of the game better. The learners showed more interest in this topic compared to the topic of ‘New Friend at School’ in Cycle 1. The main reason may be that the topic was related to their adult lives and relationships. Therefore, every learner had something to contribute to the wrap-up session.

#### 3.5.8 Findings of Cycle 2

• The lesson titled ‘Misunderstanding’ was executed using the ECP game on the computer as the second lesson. It was interactive and helped the learners to reduce learning stress due to the relevance of the topic to their real lives.
• More time and practice enabled the learners to overcome the speaking problems related to this topic in terms of its vocabulary, phrases, and pronunciation.
• The game-based learning was found to be effective in enhancing the learners' listening and speaking skills, along with their range of vocabulary.
• The learners were particularly interested and motivated to correct their pronunciation by listening to the game. This motivated them to speak using correct and intelligible pronunciation.

## 4 Results

This section summarizes the substantial findings revealed from the interview and detailed field notes maintained throughout the current study:

• The use of a computer-based game in ESL learning motivated the learners to overcome their speaking inhibition and participate in the conversations.
• The topics of interest significantly increased the learners' motivation in the game-based learning environment, and it was observed that the learning was enjoyable.
• The game improved students' confidence when they performed the ‘Misunderstanding’ conversation. This could be explained by the relevance of the topic.
• The media used in the ECP game was effective in attracting and holding the learners' attention and engaging them with the lesson.
• The rules of the game initially confused the learners, but ample time and practice made them fluent in using the game. Moreover, it was observed that a little use of the native language for giving directions increased the learners' understanding of the game.
• The applied games, which do not interest students, may lead to difficulties related to interest, motivation, executability, and understanding.
• Computer game-based learning was powerful in overcoming the learners' inhibition in speaking the target language (i.e., English).

Before and after the implementation of the action research, the points of comparison are summarized in Table 2 below:

**Table 2:** Results of experimental classes.

S/N	Activities before game-based learning	Cycle 1 After lesson 1 using the game-based learning	Cycle 2 After lesson 2 using the game-based learning
1	The learners lacked motivation to respond to the teacher's questions.	The learners were not motivated to answer the teacher's questions.	Numerous learners were willing to respond to the researcher's queries.
2	The learners were not persuaded to share their thoughts on the topic that was taught.	The learners were not convinced to express their thoughts on the taught topic.	Numerous learners were eager to express their thoughts on the topic presented in the game.
3	During the class, the learners appeared sleepy, serious, and bored.	During the class, the learners were sleepy, serious and bored.	Numerous learners were looking forward to fun learning experiences.
4	The learners' focus in the class was lacking	The learners were not very focused in the class.	Almost all learners were focused on what was being displayed in front of them.
5	It seemed as though the learners had memorized their spoken responses.	The learners seemed to have memorized what they were speaking out.	The majority of learners were still trying to memorize the video clips.
6	The learners needed a considerable amount of time to prepare their speeches.	The learners required a long time to prepare their speech.	The time allotted for preparing the talk was even shorter.
7	The pronunciation of the learners who did speak was difficult to understand.	The learners had limited vocabulary on the topic while coerced to speak.	The vocabulary of numerous learners on the topic seemed to have improved after the game was displayed.
8	The pronunciation of the learners who did speak was difficult to understand.	The learners who talked had unintelligible pronunciation.	The learners' pronunciation of the vocabulary presented had improved.

**Fig. 5:**
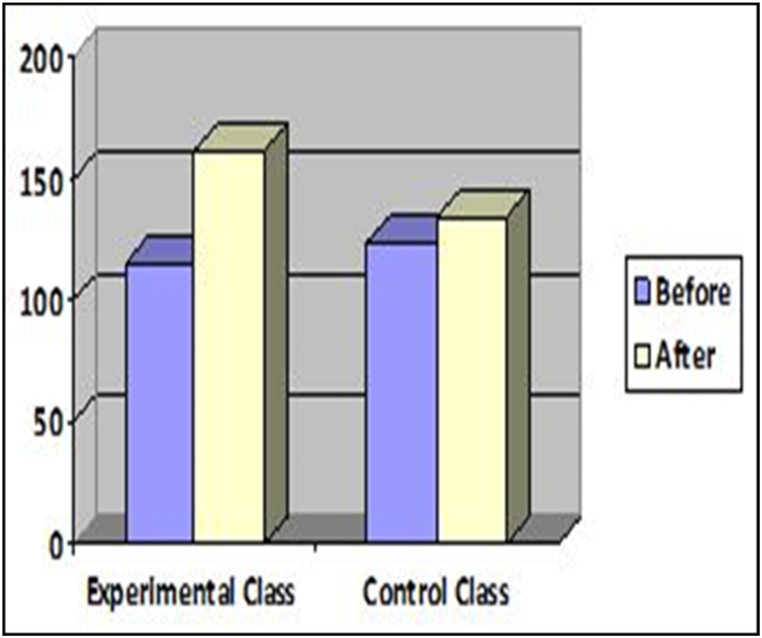
Scores for experimental class versus control class.

To assess any potential improvement in the respondents' speaking skills, a post-test was administered using the scoring rubrics developed by Purnawam et al. [27]. The scores were rated and subsequently verified by the teacher. The agreed-upon scores were deemed final. Fig. 5 displays the scores for both the control and experimental classes:

Fig. 5 illustrates that the respondents' scores in both Cycle 1 and Cycle 2 increased in the experimental class:

According to the illustration, the scores of the experimental class surpassed those of the control class. It is also shown that control class, in which no gamification was introduced, improved very little. This indicates that the introduction of the ECP game in the experimental classes enhanced the speaking skills of the learners. The improvement, as defined by the rubrics, was recorded in terms of fluency, accuracy, vocabulary, pronunciation, and knowledge.

For further statistical information and robust results, an inferential test was conducted. A paired sample *t*-test was performed to compare the possible differences between the means of experimental and control groups. Table 3 illustrates the findings of this test:

**Table 3:** T-test mean scores and standard deviation of experimental and control groups.

	Mean	Std. Dev.	*t*	Df	Sig.
	($\bar{x}$)				(2-tailed)
Experimental Group	20.00	2.02	3.48	416	0.02^a^
Control Group	14.00	2.30			

Note:

a *p* < .05.

Table 3 demonstrates that the experimental group obtained a higher mean score ($\bar{x}$ = 20.00, *SD* = 2.02) than that of the control group ($\bar{x}$ = 14.00, *SD* = 2.30). It shows that there was a significant difference between the performance of both groups with the experimental group outperforming the control group, *t* (28) = 3.48, *p* = 0.02 (*p* < .05).

Furthermore, observations suggested that computer game-based learning boosted the students' confidence, motivation, and willingness to participate in class. Therefore, the triangulation of the test results, observations, and interviews confirmed an increase in learner performance.

## 5 Discussion

This study aimed to explore how gamification contributes to enhancing English speaking skills for learners of English as a second language. Distinct from existing literature, this research developed an action plan to link digital gamification with the improvement of speaking skills. The focus was on understanding students' perceptions of how gamification through digital games supports their speaking abilities and influences their motivation. Additionally, the study proposed strategies to address students' negative attitudes toward speaking skills identified in previous research.

Gamification's primary objective is to make learning more engaging for students [31]. In pursuit of this goal, this study undertook an extensive application process, gathering rich qualitative data. It has previously been found that digital gamification heightened student interest and enhanced the effectiveness of the teaching process, a finding that aligns with the outcomes of this study [32]. However, this study diverged from others by concentrating specifically on speaking skills, recognized as crucial for communication, whereas other studies have typically focused solely on vocabulary acquisition.

To improve the speaking skills of international students enrolled in the English Intensive Course (IEC) at the UUM Language Center, the researcher conducted action research using computer game-based learning. The research was divided into three main stages, each with its own substages and specific activities aligned with the planned objectives.

The pre-research stage included observations of both the experimental and control classes to identify significant difficulties in English speaking skills, followed by a pre-test to assess the students' current speaking levels. During the research implementation stage, a traditional lesson was delivered to the control class, while the ECP game was administered to the experimental class. The post-research stage consisted of a post-test for both classes to measure any improvements in speaking skills.

The learners' speaking abilities improved significantly through the use of the ECP game, which included activities such as listening, learning new vocabulary, and speaking practice. This intervention led to notable enhancements in fluency, pronunciation, accuracy, clarity, and content. Additionally, the learners demonstrated increased interest in practicing the language.

It is to be noted that the present study has some limitations as is the case with every research. Firstly, though the data were highly representative, a larger sample could provide more chances of generalizability. Future researchers may like to investigate the gamification of language learning using a larger sample that will assist in increasing generalizability. Secondly, this study was restricted to two weeks because of the scheduled learning program of the university. Stretching the time of the intervention may help obtain a better understanding and results regarding the effect of using games in L2 learning. Lastly, this study was conducted in only one research setting (i.e., one university). Future investigations may simultaneously involve more research settings to reveal a bigger picture of how different educational settings and their students respond to L2 learning through gamification. Further, a variety of games may be used.

## 6 Implications

This action research has yielded several significant implications that can be applied to games-based learning. These implications can be summarized as follows:

### 6.1 For teachers

The findings have significant implications for L2/EFL/ESL teachers, particularly in teaching speaking skills. The results suggest that computer game-based teaching can initiate amusing learning, engage learners, and produce positive outcomes due to their willingness and interest in the content and its delivery method. Digital games, like ECP, that are conveniently available, can thus be effective in improving language learner performance.

### 6.2 For learners

Conducted in the context of university learners, including international students, the study revealed that learners could enhance their L2/EFL/ESL speaking skills using games available online or as computer apps. As adults, university learners can self-regulate their learning by immersing themselves in game-based learning. For example, ECP can be downloaded on phones and computers, and can be used as a tool for independent learning or in integrated form with general lessons in speaking.

### 6.3 For future researchers

Future researchers might expand upon this research by incorporating qualitative methods such as interviews with teachers and learners to gather perceptions of learning L2 through computer games. Additionally, they could explore the potential for improvement in L2 learner performance in contexts outside of Malaysia. Investigating the administration of computer game-based learning in a homogeneous environment where no learner is international could also be valuable. Moreover, future researchers could refine the methodology by adding relevant substages to the three main stages of action research, thereby enhancing the research approach.

## 7 Conclusion

This study has navigated the impact of utilizing computer-based games in learning English as a second language among university-level international students in Malaysia. Enhancing English speaking skills for better student engagement in academic activities is crucial for learners with diverse linguistic backgrounds. This study contends that innovative and interesting teaching methods and materials can furnish desired language learning outcomes. The study presents the empirical findings on the effects of introducing gamification in Intensive English Course classes for international students at University Utara Malaysia (UUM) who previously secured the lowest score in speaking. Using O'Brien's (1998) [22] spiral action research model, this study conducted speaking skill intervention with 15 learners in the experimental group while the control group also consisted of 15 learners. A fine-tuned two-stage roadmap of “plan, act, and observe” and “revise plan, act, and observe” was followed for introducing the ECP game for teaching and learning the selected lesson plans on socialization and other language functions. To strengthen the quantitative findings, semi-structured interviews were also conducted with 12 learners at the pre-research and post-research stages for knowing the learner perception regarding learning through gamification. The triangulated findings demonstrated that digital gamification has a significant effect on the speaking performance of the respondents. The respondents endorsed using computer-based games for language learning. The study has wide implications for stakeholders such as teachers, learners, course designers, curriculum managers, and washback strategists. The study also recommends further research actions in the field involving different computer-based games among international learners in different contexts, different age ranges, specific course learning, larger sample sizes, and different research approaches and methods.

## Ethical approval

The ethical approval of this research was issued by the Internal Research Review Board of Northern University Malaysia, also referred to as University Utara Malaysia (UUM). The request was served by the Board Head. Furthermore, the respondents’ consent was taken using a research participation consent form, and they were duly informed about their right to processual withdrawal at any time.

## Consent to publish

We, the authors, give our consent for the publication of the identifiable details, which can include a photograph(s) and/or videos and/or case history and/or details within the text of this article.

## Funding

No funding was received for this research project.

## Availability of data and materials

The study was executed in fulfilment of a university degree therefore, the availability of the raw data is under embargo for one year. However, the official repository in which the data is stored is liable to provide it for public and no cost after the period of embargo.

## CRediT authorship contribution statement

**Omar Al-Jamili:** Supervision, Project administration, Conceptualization. **Musharraf Aziz:** Writing – review & editing, Writing – original draft. **Fathey Mohammed:** Validation, Formal analysis. **Abdullah Almogahed:** Methodology, Conceptualization. **Abdulwadood Alawadhi:** Resources, Investigation.

## Declaration of competing interest

The authors declare that they have no known competing financial interests or personal relationships that could have appeared to influence the work reported in this paper.
